# Curcumin and *α*/*β*-Adrenergic Antagonists Cotreatment Reverse Liver Cirrhosis in Hamsters: Participation of Nrf-2 and NF-*κ*B

**DOI:** 10.1155/2019/3019794

**Published:** 2019-04-30

**Authors:** José Roberto Macías-Pérez, Bruno Jesús Vázquez-López, Martin Humberto Muñoz-Ortega, Liseth Rubí Aldaba-Muruato, Sandra Luz Martínez-Hernández, Esperanza Sánchez-Alemán, Javier Ventura-Juárez

**Affiliations:** ^1^Química Clínica, Unidad Académica Multidisciplinaria Zona Huasteca, Universidad Autónoma de San Luis Potosí, Ciudad Valles, SLP, Mexico; ^2^Departamento de Química, Centro de Ciencias Básicas, Universidad Autónoma de Aguascalientes, AGS, Mexico; ^3^Departamento de Morfología, Centro de Ciencias Básicas, Universidad Autónoma de Aguascalientes, AGS, Mexico

## Abstract

Liver cirrhosis is the result of an uncontrolled fibrogenetic process, due to the activation and subsequent differentiation into myofibroblasts of the hepatic stellate cells (HSC). It is known that HSC express adrenoreceptors (AR), and the use of AR antagonists protects experimental animals from cirrhosis. However, several studies suggest that the toxicity generated by metabolism of these antagonists would hinder its use in cirrhotic patients. In addition, liver fibrosis may be associated with a decrease of the antioxidant response of the nuclear factor erythroid 2-related factor 2 (Nrf-2) and the overregulation of the proinflammatory pathway of nuclear factor kappa B (NF-*κ*B). Therefore, in the present work, the capacity of doxazosin (*α*1 antagonist), carvedilol (nonselective beta-adrenoceptor blocker with alpha 1-blocking properties), and curcumin (antioxidant and anti-inflammatory compound) to reverse liver cirrhosis and studying the possible modulation of Nrf-2 and NF-*κ*B were evaluated. Hamsters received CCl4 for 20 weeks, and then treatments were immediately administered for 4 weeks more. The individual administration of doxazosin or carvedilol showed less ability to reverse cirrhosis in relation to concomitantly curcumin administration. However, the best effect was the combined effect of doxazosin, carvedilol, and curcumin, reversing liver fibrosis and decreasing the amount of collagen I (Sirius red stain) without affecting the morphology of hepatocytes (hematoxylin and eosin stain), showing normal hepatic function (glucose, albumin, AST, ALT, total bilirubin, and total proteins). In addition, carvedilol treatment and the combination of doxazosin with curcumin increased Nrf-2/NF-*κ*B mRNA ratio and its protein expression in the inflammatory cells in the livers, possibly as another mechanism of hepatoprotection. Therefore, these results suggest for the first time that *α*/*β* adrenergic blockers with curcumin completely reverse hepatic damage, possibly as a result of adrenergic antagonism on HSC and conceivably by the increase of Nrf-2/NF-*κ*B mRNA ratio.

## 1. Introduction

Liver cirrhosis is the final consequence of various chronic liver diseases [1]. This pathology is a major global health problem, because it is the 11th most common cause of death worldwide [2]. The resulting loss of hepatic architecture and functionality is attributed to the chronic inflammation, necrosis, and fibrosis. The fibrotic process is characterized by the accumulation of extracellular matrix (ECM) or scar tissue, which distorts the hepatic architecture forming fibrous bands and the subsequent development of nodules of regenerating hepatocytes [3]. The hepatic stellate cells (HSC) are responsible for initiating and spreading liver fibrosis, secreting fibrogenic factors that encourage portal fibrocytes, fibroblasts, and bone marrow-derived myofibroblasts [4]. During fibrosis process, the HSC are fully activated to a profibrogenic myofibroblastic phenotype, expressing alpha-smooth muscle actin (*α*-SMA), increasing the production of type I and III collagens, and increasing the synthesis and extracellular release of tissue inhibitors of metalloproteinase- (TIMP-) 1 and 2, which inhibit ECM degradation by inhibiting the matrix metalloproteinases (MMPs) [5, 6].

The progression through the different stages of chronic liver damage is associated to oxidative stress, inflammation, and fibrogenic molecular mechanisms, which drive to cirrhosis. The nuclear factor kappa B (NF-*κ*B) is a transcriptional regulator of the inflammatory response in liver damage, associated with proinflammatory gene expression [7]. In addition, NF-*κ*B has a broad range of functions such as influencing the survival of hepatocytes, inflammation through Kupffer cell, and activation and survival in HSC [8]. Besides, the factor nuclear factor erythroid 2-related factor 2 (Nrf-2) is an oxidative stress-sensitive transcriptional factor, associated with protection of hepatic cells against oxidative damage [9, 10].

On the other hand, several evidences have shown that fibrosis results from excessive activation of the sympathetic nervous system (SNS) [11]. In addition, the use of adrenergic receptor (AR) blockers, such as nonselective *α*-AR and *β*-AR antagonists, inhibits the growth of HSC and reduce liver fibrosis [12]. Likewise, diverse studies have shown that HSC express a variety of adrenoreceptor subtypes *α*1-AR and *β*2-AR, which can promote its activation and ECM secretion [13]. However, antiadrenergic agents are metabolized by the liver into active, inactive, or even toxic metabolites [14]. Carvedilol, a nonselective *β*-AR, and doxazosin, an *α*1-AR blocker (antihypertensive drugs), show antifibrotic effects in *in vivo* studies [15, 16]. Likewise, it was reported that carvedilol increases risks for hepatorenal syndrome and acute kidney injury, reducing transplant-free survival in cirrhotic patients with spontaneous bacterial peritonitis [17], whereas doxazosin shows hepatotoxic effects in the cirrhotic livers of experimental animals [18, 19].

Accordingly, in the present study, it was evaluated the ability to reverse liver cirrhosis by treatment with doxazosin and carvedilol, as well as the cotreatment with curcumin, looking to attenuate the toxic effects of these AR blockers. In addition, changes in Nrf-2 and NF-*κ*B mRNA expressions and in the protein expression were examined. In the present study, it was demonstrated that AR antagonists significantly reverse fibrosis, even though the livers were not completely healthy, but curcumin coadministration with each AR antagonist showed better effect. However, our results suggest that the combination of the three compounds (doxazosin, carvedilol, and curcumin) completely reverse liver cirrhosis, showing a normal cellular architecture and an appropriate hepatic function. In addition, the present article suggests for the first time that doxazosin and carvedilol increase Nrf-2 and Nrf-2/NF-*κ*B mRNA expression and the presence of these proteins in inflammatory cells, respectively.

## 2. Materials and Methods

### 2.1. Chemicals

Doxazosin, carvedilol, and curcumin were purchased from Sigma Chemical Company (St. Louis, MO, USA). Carbon tetrachloride and formaldehyde were obtained from J.T. Backer (Xalostoc, Mexico). Pentobarbital was obtained from MAVER Laboratories.

### 2.2. Experimental Animals

Male golden hamsters (*Mesocricetus auratus*, 100-150 g) were maintained on a light/dark cycle (12 : 12) and fed with Purina Rodent Chow and water *ad libitum*. All animal experiments were approved by the Animal Welfare and Research Ethics Committee of the Autonomous University of Aguascalientes (Aguascalientes, Mexico) and were conducted in accordance with institutional guidelines for care and use of experimental animals and with the national regulatory norm NOM-062-ZOO-1999. Experimental protocols were approved by the Institutional Bioethical Committee.

### 2.3. Induction of Liver Cirrhosis in Hamsters

**Figure 1:**
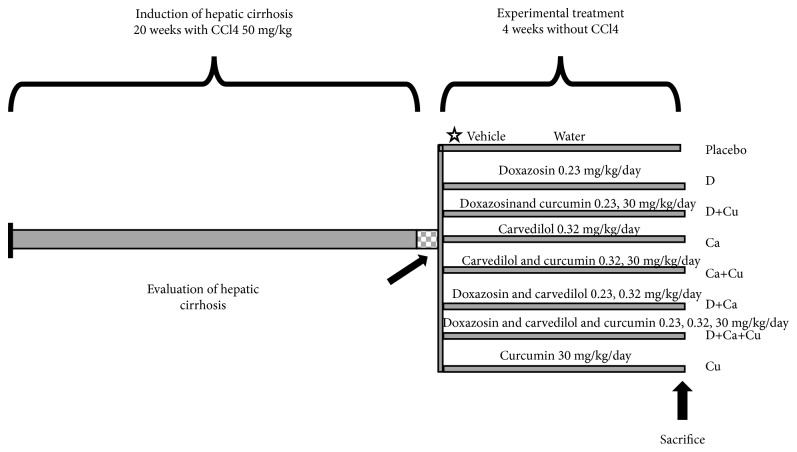
Schematic representation of *α*/*β*-AR antagonists and curcumin treatments in the CCl4-induced cirrhosis in hamster. CCl4: carbon tetrachloride; doxazosin (D group), doxazosin and curcumin (D+Cu), carvedilol (Ca group), carvedilol and curcumin (Ca+Cu), doxazosin and carvedilol (D+Ca), doxazosin, carvedilol, and curcumin (D+Ca+Cu), and Curcumin (Cu).

Fifty hamsters were included in the present protocol. The experiment began with two groups: (i) healthy animals (intact group, *n* = 5) and (ii) CCl4 treatment: the cirrhosis was induced in 45 hamsters by intraperitoneal administration of 50 mg/kg CCl4 dissolved in petrolatum, two times per week during twenty weeks (Figure 1). These cirrhotic animals were further divided in nine groups: (i) cirrhotic group: 5 animals were sacrificed at the end of the CCl4 treatment. The other 40 cirrhotic animals, after suspending CCl4 toxicity, were administered daily for 4 weeks more with the respective treatment (*n* = 5 each group); (ii) placebo group: it was administered with vehicle (0.5 ml of water, p.o.) to evaluate endogenous reversal; (iii) D group: doxazosin (0.23 mg/kg, p.o.); (iv) D+Cu group: doxazosin (0.23 mg/kg, p.o.)+curcumin (30 mg/kg, p.o.); (v) Ca group: carvedilol (0.32 mg/kg, p.o.); (vi) Ca+Cu group: carvedilol (0.32 mg/kg, p.o.)+curcumin (30 mg/kg, p.o.); (vii) D+Ca group: doxazosin (0.23 mg/kg, p.o.)+carvedilol (0.32 mg/kg, p.o.); (viii) D+Ca+Cu group: doxazosin (0.23 mg/kg, p.o.)+carvedilol (0.32 mg/kg, p.o.)+curcumin (30 mg/kg, p.o.); (ix) Cu group: curcumin (30 mg/kg, p.o.). Groups of healthy animals were included, which were treated in the same way as the cirrhotic groups, but instead of the hepatotoxic compound CCl4, they were administered with petrolatum (200 *μ*l, i.p.).

### 2.4. Sacrificed Animals

.Hamsters were anesthetized with sodium pentobarbital (50 mg/kg, i.p.), the blood was collected via cardiac puncture, and the livers were carefully dissected, freed from surrounding fatty and fibrous tissues. Liver samples were fixed in 4% neutral formalin for histological examination. Later, RNA was isolated for qPCR. Serum samples were also obtained to measure liver function markers.

### 2.5. Histological Analysis

Liver damage was evaluated whit hematoxylin/eosin (H&E) looking for architectural/morphological changes in tissue and Sirius red stain by polarized light microscopy for determination of collagen fibers deposits (type I = red, type III = green). The histological preparations were visualized using a Zeiss Axioscope 40/40 FL microscope and analyzed with the Image Pro Plus Software 4.5.1 (Media Cybernetics, Bethesda, MD, USA). Percent of fibrotic area was determined as the ratio of fibrotic area in relation to the total area of the tissue.

### 2.6. Immunohistochemical Analysis for NF-*κ*B and Nrf2

Immunohistochemistry was performed to analyze NF-*κ*B and Nrf2-positive cells in liver tissues. Briefly, liver tissue slides, 5 *μ*m thick, were incubated with primary antibodies rabbit anti-NF-*κ*B (Invitrogen 44-711G) and rabbit anti-Nrf2 (Cell Signaling12721S) diluted 1 : 100 in PBS for 12 h at 4°C; as a secondary antibody, we used a goat anti-rabbit IgG (SIGMA A0545) diluted 1 : 200 in PBS. The peroxidase activity was developed with Sigma Fast Diaminobenzidine (Sigma-Aldrich D12384); the number of NF-*κ*B and Nrf2-positive cells was counted in slides and reported as cells/mm^2^. Data was documented in a Zeiss Axioscope 40/40FL microscope (Zeiss, Oberkochen, GE) and analyzed with the Image Pro Plus Software 4.5.1 (Media Cybernetics, Bethesda, MD, USA).

### 2.7. Markers of Liver Damage

Serum samples were analyzed to determine liver damage, quantifying levels of glucose, albumin, total proteins, alanine aminotransferase (ALT), and aspartate transaminase (AST), which were measured on a spectrophotometric semiautomatic BTS-350 analyzer (Biosystems, Quezon City, Philippines).

### 2.8. Isolation of Total RNA and Reverse Transcription-Quantitative Polymerase Chain Reaction (RT-qPCR)

Total RNA was isolated from 100 mg of liver tissue of control and experimental animals with the SV Total RNA Isolation System (Promega, Madison, WI, USA), according to the manufacturer's protocol. Total RNA was quantified with a NanoDrop-2000 (Thermo Scientific, Waltham, MA, USA) and stored at -80°C until needed. Reverse transcription was performed with 1 *μ*g of total RNA with the GoScript Reverse Transcription System (Promega). Subsequently, real-time quantitative PCR was performed by using the qPCR green Master with UNG-clear (Jena Bioscience, Jena, Germany) on a StepOne apparatus (Applied Biosystems) in the following manner: 50°C for 2 min, 95°C for 45 sec, 40 cycles of 95°C for 45 sec and 60°C for 45 sec. Oligonucleotides were designed to target NF-*κ*B, Nrf-2, and *β*-actin (Nrf-2: forward: 3′ GCAAGATGGAGGAGAACGAG 5′, reverse: 5′ CCTCCAGCAGCCTAAGACAC 3′; NF-*κ*B: forward: 3′ CAGGAGCCTCAAACCTGAAG 5′, reverse: 5′ CGTCTGTGGGAGAGAAGTCC 3′) and actin as reference control (forward: 3′GCCCAGAGCAAGAGAGGTAT5 ′, reverse: 5′ CACGCAGCTCGTTGTAGAAG 3′). Relative expression levels were normalized against *β*-actin as an internal housekeeping gene, and differences were determined by employing the ΔΔCt relative method.

### 2.9. Statistical Analysis

GraphPad Prism 5.00 software was employed for statistical analysis and figures. Data were expressed as the mean values ± standard error of each group. Significant differences between mean values were assessed by using the two-way analysis of variance followed by Tukey's test. Differences were considered statistical significant at *p* < 0.05.

## 3. Results

### 3.1. Regression of Liver Cirrhosis with *α*/*β*-AR Antagonists and Curcumin Treatments

**Figure 2:**
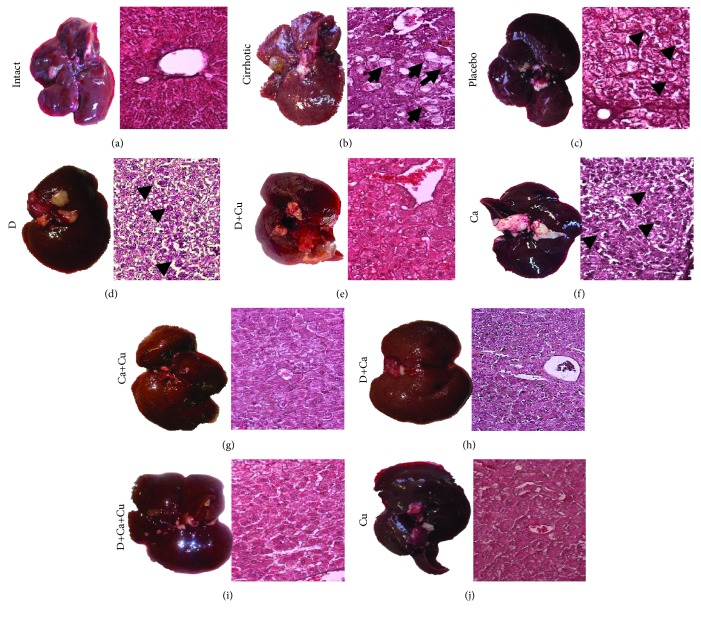
Macroscopic and histological H&E staining observations of the effect of *α*/*β*-AR blockers and curcumin on CCl4-induced liver cirrhosis in hamster. Representative pictures of the healthy livers (intact group), the livers of animals with cirrhosis induced (cirrhotic group) and, after the cirrhosis, treated with water (placebo), doxazosin (D), carvedilol (Ca), curcumin (Cu), doxazosin and carvedilol (D+Ca), doxazosin and curcumin (D+Cu), carvedilol and curcumin (Ca+Cu), and the combination of doxazosin, carvedilol, and curcumin (D+Ca+Cu). The H&E staining shows in the cirrhotic group: steatosis (black arrow) and necrosis (asterisk); in the placebo group: ballooning (arrowhead) and necrosis (asterisk); and in the D and Ca groups: ballooning (arrowheads). Magnification, 20x.

Macroscopic and histopathological examinations of the livers indicated that *α*/*β*-AR antagonists and curcumin treatments reverse liver damage induced by CCl4 (Figure 2). The intact group evidenced livers with normal aspect, dark-browned color, and smooth surface, as well as the H&E staining showed classic architecture of hepatic lobules with cords of hepatocytes arranged radially around the central vein (Figure 2(a)). The cirrhotic group evidenced fibrotic liver, characterized by surface granulations on the entire liver known as regenerative nodules, and H&E staining exhibited a damaged liver architecture, represented by a large number of hepatocytes with macro- and microvesicular steatosis and necrotic cells (Figure 2(b)). The placebo group presented opaque and pale livers with respect to intact animals, as well as regenerative nodules were observed; the hepatocytes showed ballooning and nuclei with karyorrhexis and karyolysis (Figure 2(c)). The livers of groups D and Ca showed less liver damage with the presence of pyknotic hepatocytes and with ballooning in relation to the placebo group (Figures 2(e) and 2(f)). Though, the liver of the D+Cu and Ca+Cu groups showed less surface granulation and smaller and fewer regenerative nodules in relation to groups D and Ca, respectively (Figures 2(e) and 2(g)). The D+Ca group showed high inflammatory infiltration with some pyknotic nuclei and alterations in the hepatic architecture compared to the intact group. On the other hand, the animals that received the *α*/*β*-AR antagonists with curcumin (D+Ca+Cu group) showed a morphology similar to the intact group.

### 3.2. Regression of Liver Fibrosis with *α*/*β*-AR Antagonists and Curcumin Treatments

**Figure 3:**
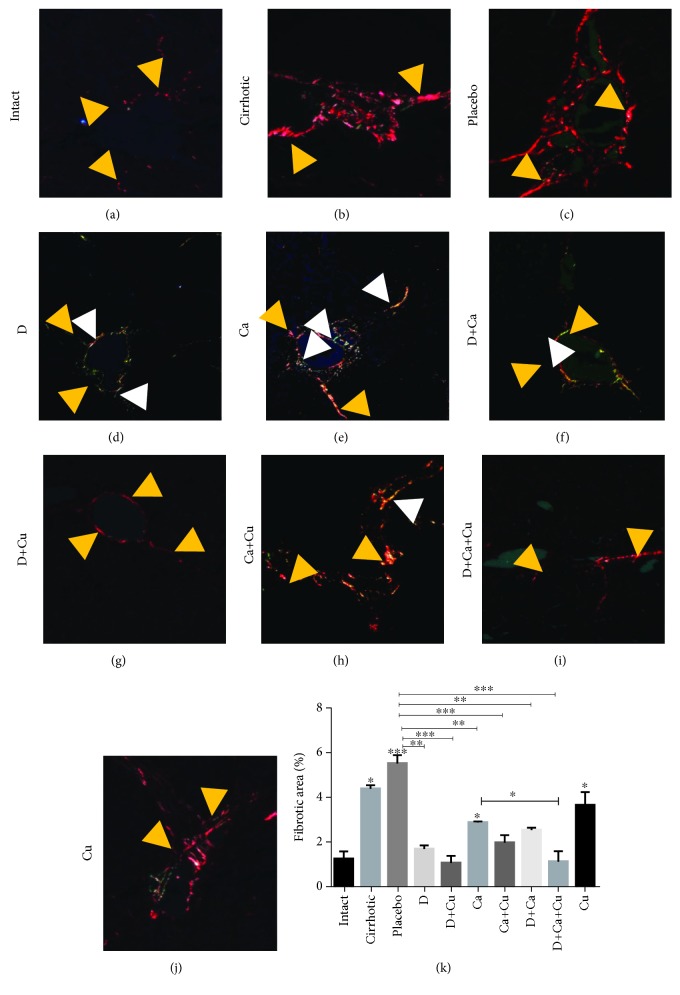
Effect of *α*/*β*-AR blockers and curcumin in the deposition of type I and III collagens in cirrhotic hamsters. For identification of collagen fiber, polarized light was used. Representative pictures of (a) intact group, (b) cirrhotic animals, and cirrhotic animals treated with (c) water (placebo group), (d) doxazosin (D group), (e) carvedilol (Ca group), (f) doxazosin and carvedilol (D+Ca), (g) doxazosin and curcumin (D+Cu), (h) carvedilol and curcumin (Ca+Cu), (i) doxazosin, carvedilol, and curcumin (D+Ca+Cu group), and (j) curcumin (Cu). Type I collagen: yellow arrowhead; type III collagen: white arrowhead. Magnification, 20x. (k) Percentage of fibrotic area was determined by measuring the amount of fibrotic area with respect to whole field area. Data is presented as mean ± SEM (*n* = 5 each group). In cirrhotic, placebo, and Ca and Cu groups vs. intact: ^∗^*p* < 0.05, ^∗∗∗^*p* < 0.001. In D+Ca+Cu vs. Ca: ^∗^*p* < 0.05. In D, D+Cu, Ca, Ca+Cu, D+Ca, D+Ca+Cu vs. placebo: ^∗∗^*p* < 0.01 and ^∗∗∗^*p* < 0.001.

Fibrotic process was evaluated with Sirius red staining evidencing accumulation of type I and III collagens (Figure 3). The intact group showed normal appearance of collagen (Figure 3(a)). The cirrhotic group evidenced large amounts of type I collagen, and the placebo group displayed thickened fibers and a greater distribution through the liver tissue, mainly around the centrilobular veins and portal triads (Figures 3(b) and 3(c), respectively). In the D group, significant thinning of collagen fibers and increase in type III collagen were observed in relation to type I (Figure 3(d)). The D+Cu group exhibited a decrease in type I collagen in the portal vein and was not observed type III collagen (Figure 3(g)). On the other hand, the treatment of Ca group displayed type I collagen plaques distributed around the portal veins, with some septa of type I and III collagens surrounding regenerative nodules (Figure 3(e)). The Ca+Cu group displayed greater thickness of type I and III collagen plaques forming regeneration nodules (Figure 3(h)) compared to the Ca group. The livers of the D+Ca group exhibited lower amount of type I and III collagens (Figure 3(f)) compared to the Ca group. Finally, the D+Ca+Cu group showed only a little amount of type I collagen with similar appearance to the intact group (Figure 3(i)). Compared to the other treatments, the Cu group showed greater amount of type I collagen in the portal triad with thin septa (Figure 3(j)). The percentages of the fibrotic areas were increased in the cirrhotic and placebo groups and were significantly reduced in cirrhotic hamsters treated with *α*/*β*-AR antagonists. However, the combination of *α*/*β*-AR antagonists with curcumin reduced the percentage of type I collagen closely to the levels of the intact group (Figure 3(k)).

### 3.3. Immunohistochemistry for NF-*κ*B and Nrf2

**Figure 4:**
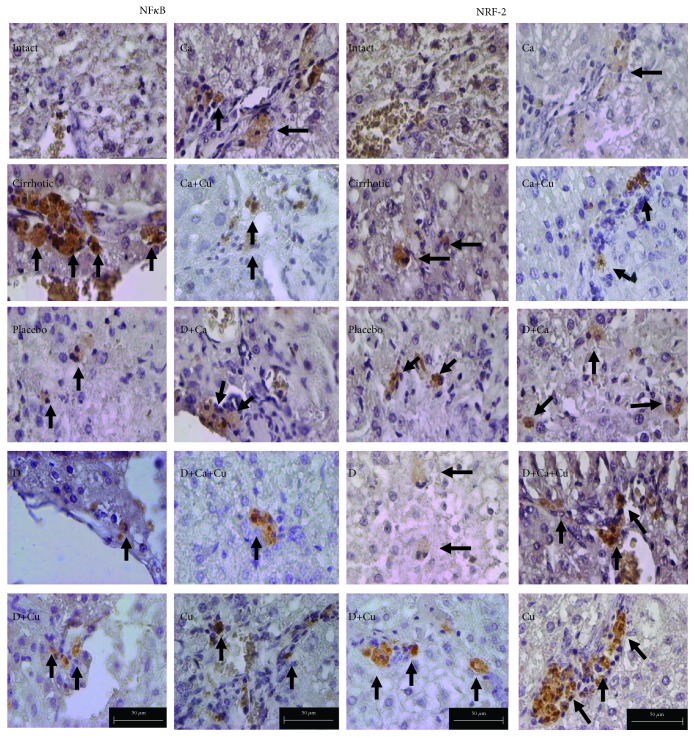
Detection of NF-*κ*B and Nrf2 in liver tissue slides by immunohistochemistry: intact, cirrhotic, placebo, doxazosin (D), doxazosin more curcumin (D+Cu), carvedilol (Ca), carvedilol more curcumin (Ca+Cu), doxazosin more carvedilol (D+Ca), doxazosin more carvedilol more curcumin (D+Ca+Cu), and finally curcumin (Cu). Positive cells to NF-*κ*B and Nrf2 are indicated by arrows (×400).

**Figure 5:**
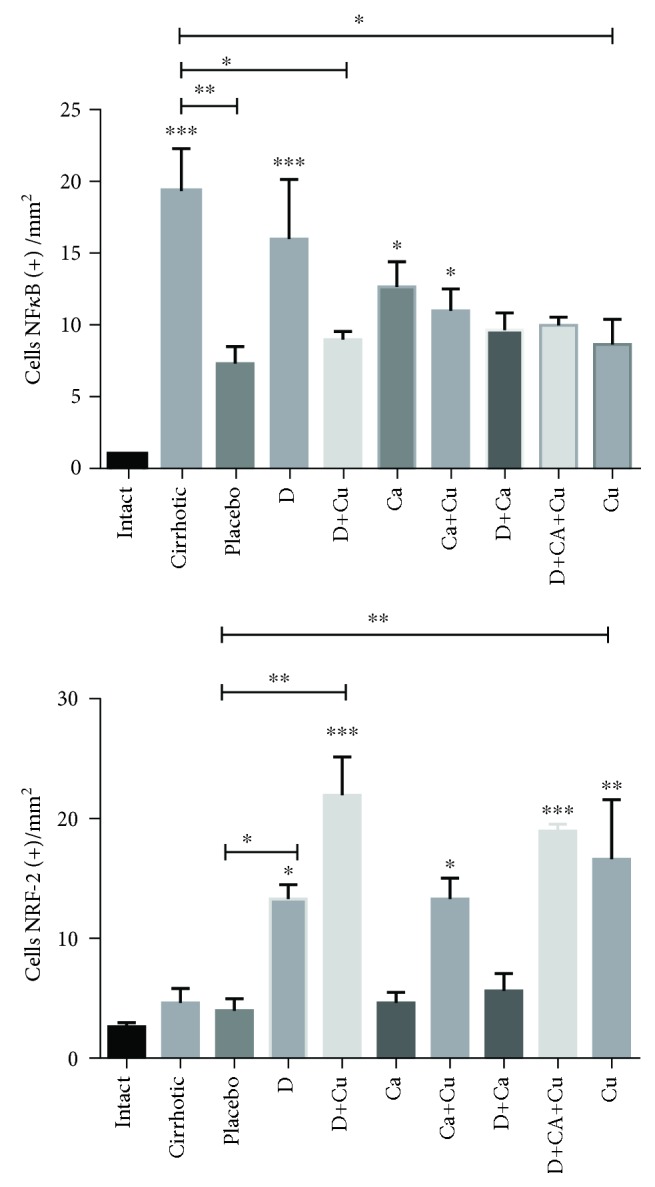
Quantification of the positive cells to NF-*κ*B and Nrf2 in slices of liver samples of the different analyzed groups.

We analyze the presence of the transcription factors NF-*κ*B and Nrf2 in the livers of hamster of different groups; in control animals, there is no presence of the Nrf2 protein or phosphorylated NF-*κ*B; on the other hand, the cirrhotic group evidenced the positivity to NF-*κ*B; on the contrary, there were few positive cells to Nrf2. However, the placebo group displayed less positivity to NF-*κ*B and similar expression of Nrf2. In the D group, significant augmenting of positive cells to NF-*κ*B and Nrf2 was observed. The D+Cu group exhibited a decrease in positive cells to NF-*κ*B and increment in positive cells to Nrf2. On the other hand, the treatment of the Ca group displayed more positive cells to NF-*κ*B with no difference to the cirrhotic group; however, the number of positive cells to Nrf2 was decreased. The Ca+Cu group showed similar number of positive cells to NF-*κ*B than the Ca group; interestingly, there were more positive cells to Nrf2. The livers of the D+Ca group exhibited lower amount of Nrf2 similar to the Ca group. Finally, the D+Ca+Cu group showed a lesser number of positive cells to NF-*κ*B and an increment of cells positive to Nrf2. The Cu group showed similar number of positive cells to both transcription factors to the D+Ca+Cu group (Figures 4 and 5).

### 3.4. Recovery of Liver Function in Cirrhotic Hamster Treated with *α*/*β*-AR Antagonists and Curcumin

**Figure 6:**
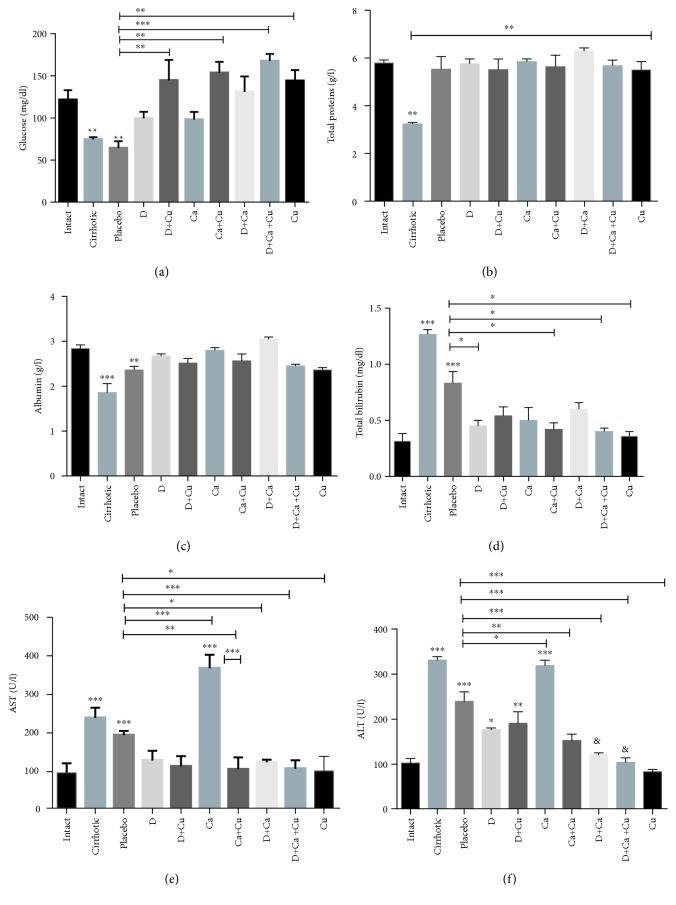
Evaluation of liver function in cirrhotic hamster treated with *α*/*β*-AR antagonists and curcumin. The serum levels of (a) glucose, (b) total proteins, (c) albumin, (d) total bilirubin, (e) AST, and (f) ALT were used as markers of liver damage. The healthy livers (intact group), the livers from animals with cirrhosis induced (cirrhotic group) and, after the cirrhosis, treated with water (Placebo), doxazosin (D), carvedilol (Ca), curcumin (Cu), doxazosin and carvedilol (D+Ca), doxazosin and curcumin (D+Cu), carvedilol and curcumin (Ca+Cu), and the combination of doxazosin, carvedilol, and curcumin (D+Ca+Cu). Data is presented as mean ± SEM (*n* = 5 each group). In glucose: D+Cu, Ca+Cu, D+Ca+Cu, and Cu groups vs. placebo, ^∗∗^*p* < 0.01 and ^∗∗∗^*p* < 0.001. In total proteins: mean values vs. cirrhotic and cirrhotic vs intact, ^∗∗^*p* < 0.01. In albumin: mean values vs. intact, ^∗∗^*p* < 0.01 and ^∗∗∗^*p* < 0.001. In total bilirubin: cirrhotic and placebo vs. intact: ^∗∗∗^*p* < 0.001. D, Ca+Cu, D+Ca+Cu, and Cu vs. placebo, ^∗^*p* < 0.05. In AST: cirrhotic, placebo, and Ca vs. intact, ^∗∗∗^*p* < 0.001. Ca, Ca+Cu, D+Ca, D+Ca+Cu, and Cu vs. placebo, ^∗^*p* < 0.05, ^∗∗^*p* < 0.01, and ^∗∗∗^*p* < 0.001. Ca+Cu vs. Ca, ^∗∗∗^*p* < 0.001. In ALT: cirrhotic, placebo, D, D+Cu, and Ca vs. intact, *p* < 0.05, ^∗∗^*p* < 0.01, and ^∗∗∗^*p* < 0.001. Ca, Ca+Cu, D+Ca, D+Ca+Cu, and Cu vs. placebo, *p* < 0.05, ^∗∗^*p* < 0.01, and ^∗∗∗^*p* < 0.001.

The serum levels of glucose, total proteins, albumin, total bilirubin, AST, and ALT were used as markers of liver damage (Figure 6). Glucose decreased significantly in the placebo and cirrhotic groups as compared to intact animals; the treatments D, Ca, and D+Ca increase glucose without reaching the normal levels, and all groups treated with curcumin reach the normal levels (Figure 6(a)). Total proteins were reduced only in the cirrhotic group, and the rest of the groups treated were complete recovery (Figure 6(b)). Albumin was significantly decreased in the cirrhotic group, whereas the placebo exhibited a slight recovery compared to the complete recovery of the rest of the treated groups (Figure 6(c)). High concentrations of total bilirubin were observed in the cirrhotic and placebo groups, the most notable being in the cirrhotic group, which showed a statistically significant difference in comparison with all the other treatments performed (Figure 6(d)). AST values increased in the cirrhotic group and decreased in the placebo group without reaching normal levels; the other treatments returned to normal levels, with an exception of the Ca group, which presented a significant increase in relation to placebo treatment (Figure 6(e)). ALT values were increased in the cirrhotic and Ca groups; placebo group reduced this parameter in relation to cirrhotic animals, while D and D+Cu reduced ALT levels significantly in relation to the cirrhotic group but not to placebo, not reaching the normal levels. Finally, Ca+Cu, D+Ca, and D+Ca+Cu returned to normal levels this marker (Figure 6(f)).

### 3.5. Regulation of Nrf-2 and NF-*κ*B mRNA Expression During the Resolution of Cirrhosis Process

**Figure 7:**
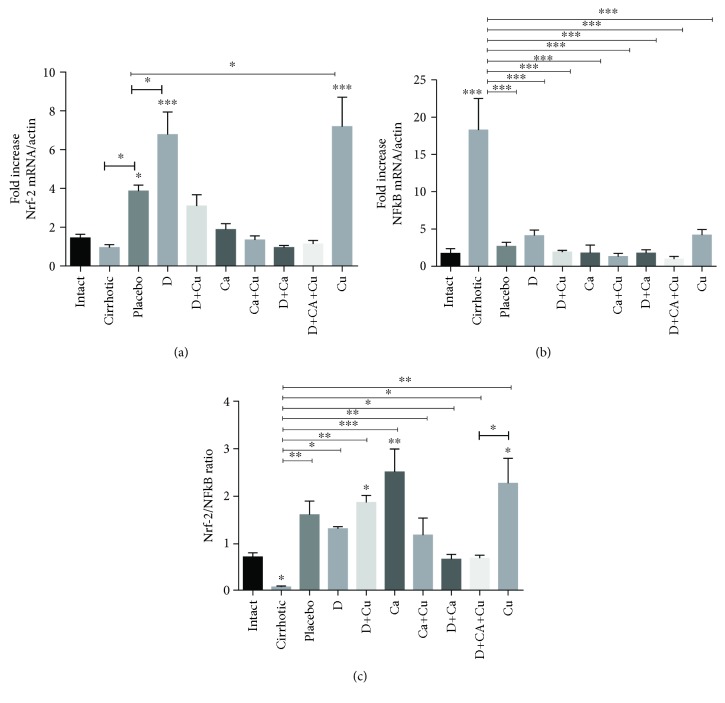
Evaluation of the effect of *α*/*β*-AR antagonists and curcumin on expression of the NF-*κ*B and Nrf-2 in the regression of cirrhosis. (a) Evaluation of the Nrf-2 mRNA. (b) Evaluation of the NF-*κ*B mRNA expression. (c) Evaluation of Nrf-2/NF-*κ*B ratio. The healthy livers (intact group), the livers of animals whit cirrhosis induced (cirrhotic group) and, after the cirrhosis, treated with water (placebo), doxazosin (D), carvedilol (Ca), curcumin (Cu), doxazosin and carvedilol (D+Ca), doxazosin and curcumin (D+Cu), carvedilol and curcumin (Ca+Cu), and the combination of doxazosin, carvedilol, and curcumin (D+Ca+Cu). Data is presented as mean ± SEM (*n* = 5 each group). In Nrf-2 mRNA/actin: placebo, D, and Cu vs. intact, ^∗^*p* < 0.05, ^∗∗∗^*p* < 0.001. Placebo vs. cirrhotic and D and Cu vs. placebo, ^∗^*p* < 0.05. In NF-*κ*B mRNA/actin: cirrhotic vs. intact, ^∗∗∗^*p* < 0.001. Mean values vs. cirrhotic group, ^∗∗∗^*p* < 0.001. In Nrf-2/NF-*κ*B ratio: D+Cu, Ca, and Cu vs. intact, ^∗^*p* < 0.05, ^∗∗^*p* < 0.01. Mean values vs. cirrhotic, ^∗^*p* < 0.05, ^∗∗^*p* < 0.01, and ^∗∗∗^*p* < 0.001. Cu vs. D+Ca+Cu, ^∗^*p* < 0.05.

The transcriptional factor NF-*κ*B controls the transcription of genes for a family of proteins involved in the response of oxidative stress and toxicity; an increase in the transcription levels of this factor is indicative of cell damage and activation of proinflammatory cytokines. On the other hand, when the transcription factor Nrf-2 is translocated to the nucleus triggers the expression of enzymes and antioxidant compounds, high levels of this factor indicate a protection of liver parenchyma [7, 20]. Therefore, in the present work, Nrf-2 and NF-*κ*B mRNA expressions were examined (Figure 7). Our results showed high levels of Nrf-2 mRNA expression in the placebo group in relation to the cirrhotic group, while the D and Cu groups showed a significant increase in relation to placebo, and the other groups were closer to normal levels, although D+Cu showed a tendency to increase (Figure 7(a)). On the other hand, NF-*κ*B increased only in the cirrhotic group, while the rest of the groups did not show significant changes with respect to the intact group (Figure 7(b)). The Nrf-2/NF-*κ*B mRNA ratio expression was calculated to get a closer approximation to the relationship between these two factors in the regression process of cirrhosis. Our results indicate a reduction of this ratio in the cirrhotic group. In addition, our results also showed a significant elevation of this ratio in the D, D+Cu, Ca, Ca+Cu, D+Ca, and Cu groups with respect to the cirrhotic group although no to the placebo, and the Ca, D+Cu, and Cu treatments significantly increased the Nrf-2/NF-*κ*B ratio in relation to the intact group (Figure 7(c)).

## 4. Discussions

In the present work, cirrhosis was induced by chronic administration of CCl4 in hamster, and the macroscopic and microscopic observations together with the markers of liver damage show cirrhotic animals with necrosis and fibrosis and lost hepatic functionality, whereas the placebo group did not return neither to characteristic cellular morphology nor to normal biochemical levels (glucose, albumin, total bilirubin, AST, and ALT). These alterations during the induction and establishment of hepatic cirrhosis with CCl4 in a hamster model are consistent with previous work [16].

Curcumin is a phenolic compound with powerful antioxidant and anti-inflammatory activities [21]. Several experimental protocols have shown that curcumin possesses hepatoprotective properties for a wide variety of liver pathologies, through various cellular and molecular mechanisms [22]. Those mechanisms include suppressing on lipid perodixation and PI3K/Akt and HSC activation, downregulating the NF-*κ*B activation, ameliorating cellular responses to oxidative stress, and upregulating the expressions of Nrf-2 and HO-1 mRNAs [22–24]. In the present work, curcumin (30 mg/kg, p.o.) was used at a dose lower than that used to reduce liver fibrosis (100 mg/kg, p.o.) [25, 26], with the purpose of attenuating the damage generated by metabolism of *α*/*β*-AR blockers, creating an environment free of oxidative stress and inflammation with the probable activation of Nrf-2 and inhibition of NF-*κ*B. In the present work, it was demonstrated that curcumin (30 mg/kg) did not reverse liver fibrosis, whereas it has been reported that curcumin (100 mg/kg) prevents and reverses cirrhosis induced by bile duct obstruction or CCl4 in rats [27]. Our results show that regression of liver fibrosis was observed with doxazosin (0.23 mg/kg) and carvedilol (0.32 mg/kg). At these doses, doxazosin significantly returned to normal-level glucose, total proteins, albumin total, bilirubin, and AST, but ALT was only partially reduced. On the other hand, carvedilol showed normal glucose, total proteins, albumin, and total bilirubin, but AST and ALT were increased. These results suggest that the livers of hamsters treated with doxazosin and carvedilol still have hepatocellular damage, possibly derived from its extensive hepatic metabolism, perhaps generating toxic intermediates [28]. Carvedilol is metabolized by cytochrome P450 system, mainly by CYP1A2, CYP2E1, CYP2C9, CYP3A4, and CYP2D6, while doxazosin does not undergo cytochrome P450 metabolism [29]. A study in a rat model provides evidence that carvedilol (10 mg/kg) ameliorates oxidative stress, lipid peroxidation, inflammation, and liver fibrosis by decreasing NF-*κ*B expression and HSC activation [15]. However, other research in a hamster model showed that doxazosin (1.0 mg/kg) and carvedilol (1.2 mg/kg) have antifibrotic activity by AR antagonism on HSC and by amelioration of transforming growth factor-*β* (TGF-*β*) secretion; however, at these doses, the cellular architecture and liver function are not completely restored, suggesting a possible toxic effect of adrenergic blockers [16, 19]. Several works have suggested the need to evaluate and modify doses of AR antagonists in cirrhotic patients, because their use in standard doses increases their toxicity and mortality in patients [30, 31]. Therefore, in this work, the AR antagonists were used at sublow doses, reducing its toxicity, as well as concomitant used with curcumin as a hepatoprotector, resulting in the complete reversal of cirrhosis.

Interestingly, the amelioration of liver fibrosis may be associated with the activation of Nrf-2 and the inhibition of NF-*κ*B [32]. Furthermore, Nrf-2 activation inhibits the nuclear translocation of NF-*κ*B via upregulation of hemeoxygenase-1 (HO-1) [33]. Compelling evidence suggested that curcumin is an Nrf-2 activator [23], whereas Nrf-2 knockdown abolished the protective effect of curcumin [34]. Likewise, it has been reported that curcumin protects the liver from damage through activation of Nrf-2 and inhibition of NF-*κ*B [35]. Our results showed that curcumin (30 mg/kg) significantly increased the protein and mRNA expression of Nrf-2 and reduced NF-*κ*B protein and mRNA expression. This suggests that cotreatment with curcumin increases the expression of the mRNA gene for Nrf-2 and promotes its activation, since the phosphorylated protein was found in different inflammatory cells in situ in the experimental animals. Noteworthy, carvedilol increased the Nrf-2/NF-*κ*B ratio as compared with the intact group, suggesting for the first time in the reversion of liver cirrhosis an additional molecular mechanism to its antioxidant properties and its effect as an adrenergic antagonist [36]. Other studies indicate that carvedilol protects neuronal cells, activating the Nrf-2 signaling pathway [37, 38]. However, doxazosin never has been linked to Nrf-2, but our results show that doxazosin increases Nrf-2 mRNA expression, and the combination with curcumin increased also Nrf-2/NF-*κ*B ratio as compared with intact animals, suggesting that doxazosin may also influence the expression of Nrf-2. Coadministration of doxazosin, carvedilol, and curcumin did not show significant changes in the expression of Nrf-2 and NF-*κ*B, possibly due to a complete reversal of liver damage, so there is no inflammation, and therefore, it is not necessary to increase antioxidant activity of Nrf-2, showing values similar to the intact group. The transcriptional factor Nrf-2 is highly expressed in the detoxification organs such as the liver, because it aids in the detoxification and elimination of potentially harmful exogenous chemicals and their metabolites [9]. In addition, Nrf-2 is essential in the cellular cycle of regenerating the hepatocyte and the modulation of apoptotic processes during liver regeneration [39], as well as it has been implicated in the regression of liver fibrosis [40]. Other studies have shown that Nrf-2 deficiency impairs liver regeneration [40, 41].

## 5. Conclusions

Our results suggest that the simultaneous use of curcumin and *α*/*β*-AR blockers (doxazosin and carvedilol) is an effective treatment to reverse the cirrhosis process in the chronic liver damage model induced by CCl4 in hamsters. The present article suggests for the first time that doxazosin and carvedilol and curcumin increase the Nrf-2 and Nrf-2/NF-*κ*B mRNA and protein expression, respectively. Curcumin in combination with *α*/*β*-AR blockers can be a new alternative to reverse liver damage in cirrhotic patients.

## Data Availability

The data that support the findings of this study are available from the corresponding author, JVJ, upon reasonable request.
